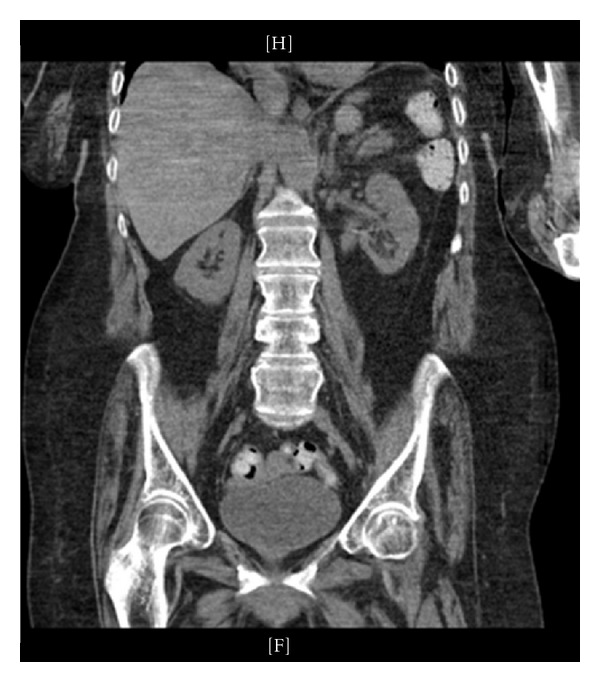# Case Report: Successful Staged Ureteroscopic Treatment of a 5 cm Staghorn Renal Calculus

**DOI:** 10.1155/2012/873069

**Published:** 2012-02-25

**Authors:** Joseph M. Ciccone, J. Clinton McCabe, Robert C. Eyre

**Affiliations:** ^1^Beth Israel Deaconess Hospital-Needham, MA 02492, USA; ^2^Harvard Medical School, Boston, MA 02115, USA; ^3^College of the Holy Cross, Worcester, MA 01610, USA

## Abstract

It is widely accepted that percutaneous nephrostolithotorny (PCNL) is the standard of choice for the removal of large staghorn renal calculi. Although data exists supporting a stagad ureteroscopic as an alternate treatment for stones up to 3 cm in select patients, little data exists to support a ureteroscopic approach for stones as large as 5 cm. We present a case of a 68 year old female with a 5 cm staghorn renal calculus managed successfully with a staged ureteroscopic approach. A staged ureteroscopic approach can be effective in treating stones as large as 5 cm.

## 1. Introduction

It is widely accepted that percutaneous nephrostolithotomy (PCNL) is the standard of choice for the removal of large staghorn renal calculi. However, there can be specific factors in a minority of patients that make alternate techniques such as ureteroscopy more favorable for the removal of large staghorn calculi.

Advances in PCNL have made it the most effective method to remove large staghorn calculi. Such advances include increasing expertise of the urologists performing the procedure, the maturation of the instrumentation, and the ability to maintain multiple tracts and perform multistaged procedures. [1]. These advances have led to a decrease in both operative time and postoperative complications and a stone clearance rate of ninety-three percent. [1]. However, the complication rate for PCNL remains fairly high at approximately eight percent. [2]. Complications from PCNL can be devastating, including disruption of the pelvicalyceal system, bowel perforation, and hemorrhaging requiring transfusion or angioembolization,. [1, 3]. PCNL requires that the patient be in the prone position. If the patient has anatomic or physical limitations that prevent prone positioning, ureteroscopy may be a reasonable alternative to open surgery.

The evolution of flexible ureteroscopes has allowed an ease of access and excellence of visualization which has helped make ureteroscopy a common procedure for most urologists. The increasing use of laser technology and electrohydraulic lithotripsy has made ureteroscopy an efficient means of treating even large renal calculi, with a stone-free rate of about eighty-seven percent in these cases [3, 4]. While ureteroscopy may have a slightly lower stone-free rate than PCNL, it also has a lower morbidity and overall complication rate, at about 2 percent [3, 4]. Since staghorn calculi are often colonized with multiple bacterial strains, all endoscopic treatments, particularly those performed via a retrograde approach, carry with them the risk of potentially lethal septic shock. Nevertheless, we feel that urosepsis and other hazards can be mitigated with a staged approach to the procedure and the proper antimicrobial precautions.

## 2. Case

**Figure 1:**
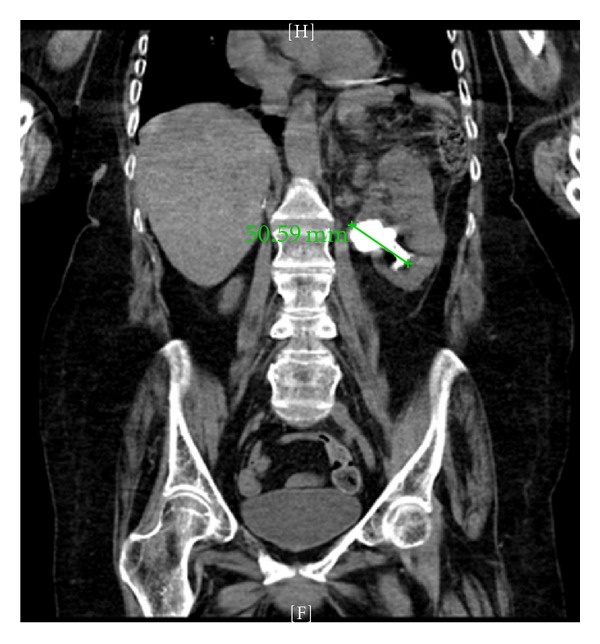


A 68-year-old wheelchair-bound female with a history of cerebral palsy and severe contractures of the upper and lower extremities presented to the emergency department with a chief complaint of fever to 100.5 F, general malaise, and dysuria. Work-up revealed grossly infected urine and a white blood cell count of 14,000. A CT scan showed a 4.8 to 5.0 cm left staghorn renal calculus with branching into the calyces (Figure 1). The patient responded well to intravenous ceftazidime and fluid resuscitation. A clean catheterized urine culture grew pan-sensitive *E. coli* and she was discharged on a 14-day course of ciprofloxacin. Prior to outpatient urologic follow-up, her symptoms recurred in a similar manner. Ceftriaxone was started empirically and a urine culture again grew *E. coli*, this time with increasing resistance and well as methicillin-resistant staph aureus. The patient was discharged on oral cefpodoxime, again for a two-week course.

Given the failure of suppressive therapy and the presence of recurrent, resistant, polymicrobial infections in the setting of a large stone nidus, we felt that removal of the stone was indicated. Stone dissolution therapy was declined in the face of such a large stone and in the setting of rapidly recurrent infection. A variety of surgical options were therefore considered. Extracorporeal shockwave lithotripsy was not felt to be an option given a stone of this size. Anatrophic nephrolithotomy and open pyelolithotomy were not technically feasible because of the patient's severe anatomic deformities. Particular consideration was given to PCNL, our treatment of choice for stones of this type. Unfortunately her body habitus again proved limiting as we were unable to place her safely into the prone or semiprone position. As an alternative, we elected to perform staged ureteroscopy and lithotripsy.

**Figure 2:**
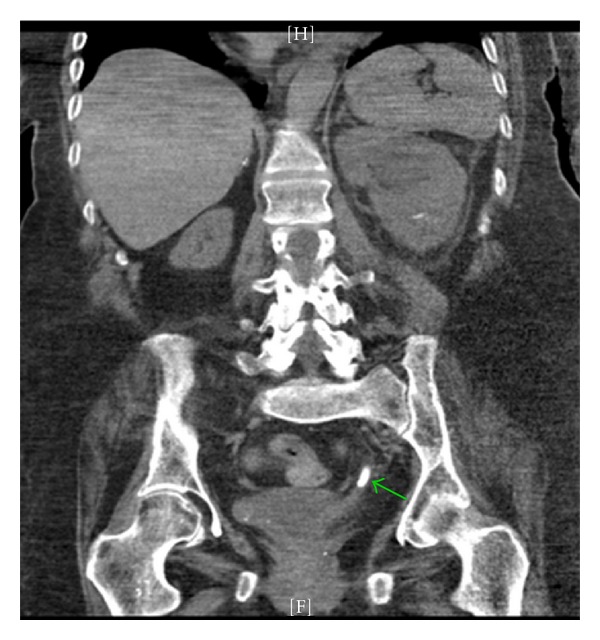


Intravenous vancomycin and gentamicin were started 24 hours prior to surgery. Stage I flexible ureteronephroscopy was carried out via a retrograde approach through a 36 cm access sheath. The 270 nanometer holmium laser fiber was used to begin fragmentation of the stone. The composition of the stone was of soft to intermediate density, and we did not feel that a larger fiber or electrohydraulic lithotripsy (EHL) was required. Lithotripsy was concluded electively after one hour and 15 minutes and a 6 French, 24-centimeter double-J ureteral stent and 18 French urethral catheter were placed. She would maintain a urethral catheter until the conclusion of her treatment. Several hours postoperatively the patient was noted to be tachycardic to 100 beats per min and with a temperature of 100.4. Vancomycin was continued, and ceftrizone begun in lieu of gentamicin. She was observed closely with prompt resolution of the tachycardia and fever, and her symptoms were attributed to transient bacteremia from surgical instrumentation. A urine culture obtained at time of surgery grew *Enterococcus faecalis* and *Pseudomonas aeruginosa*. Antibiotic coverage was switched to IV Zosyn, which was extended and carried through a second-stage ureteroscopy two weeks later and subsequent stent removal two weeks after that. Total lithotripsy time for Stage II was again roughly 1 hour and 15 minutes, and at this point the stone was felt to be fragmented into pieces that would spontaneously pass postoperatively. Two weeks after Stage II, the stent was removed. One week after stent removal, the patient developed left flank pain and malaise. A CT showed a single small obstructing distal ureteral fragment (Figure 2). In an effort to drain the infected, obstructed kidney nephrostomy was performed. Urine culture aspirated upon placement of the nephrostomy now showed Klebsiella sensitive to Ciprofloxacin. After quick resolution of symptoms, a final look ureteroscopy was performed which confirmed passage of the distal ureteral stone as well as the vast majority of the fragments in the collecting system. One final small 5-6 mm fragment was treated quickly and easily using the laser. A stent was replaced. Ciprofloxacin was continued throughout this procedure and through the stent removal two weeks later. Follow-up imaging confirmed adequate treatment of the stone (Figure 3). The nephrostomy and urethral catheter were then removed. Cipro was weaned over the next 3 weeks. At 6 months there have been no significant sequela and the patient's overall mental status and functional status are significantly improved since removal of the chronically infected stone.

## 3. Discussion

We feel that the treatment of choice for large staghorn renal calculi remains PCNL. Nonetheless, in select patients consideration must be given to a staged ureteroscopic approach as primary treatment. Ample data supports the use of staged ureteroscopy for stones up to 2 to 3 cm in size. However, for stone diameters between 3 and 5 cm, there is little data to support such an approach. Given the 3-dimensional nature of stones, we see a drastic rise in stone volume at diameters over 3 cm, making the prospect of successful ureteroscopic approach seem daunting, especially when considering the possibility of multiple procedures. However, one must remember that a second look ureteroscopy is often required to clear out residual stone fragments even when PCNL is used as primary treatment. For this reason we do not view a staged primary ureteroscopic treatment to impose a significant enough anesthetic risk as to preclude it from being considered in carefully selected patients and in the appropriate hands. We feel that successful primary ureteroscopic treatment is possible by using sound surgical principles and careful pre and postoperative management techniques.

Preoperatively, careful consideration should be given to previous culture data in order to properly select antimicrobial prophylaxis. We advise obtaining cultures through either sterile catheterization or aspiration of the bladder or kidney preoperatively, and also obtaining intraoperative cultures whenever possible. Antibiotic management should be carried out with an understanding that staghorn stones could harbor a variety of bacteria and antimicrobial resistance not always accurately reported on culture.

Operatively, retrograde irrigation should be limited during the surgical procedure. We routinely use the pulsatile pump irrigator sparingly and advocate against continuous high-pressure flow systems to mitigate against pyelovenous seeding of bacteria. Third, we suggest that total operative time be limited to under 90 minutes of lithotripsy per stage. We also suggest that careful consideration be given to the consistency of the stone at the onset of fragmentation. Should the stone be refractory to quick obliteration by laser, the use of electrohydraulic lithotripsy should be considered by the surgeon skilled in its use. For stones refractory to EHL, one should reconsider the overall feasibility of a successful ureteroscopic procedure in general. Finally, close attention should be paid to the patient's intraoperative vital signs, and the case be terminated after immediate stent placement for any signs of urosepsis including hypotension, tachycardia, and fever.

Postoperatively, our patient developed an obstruction from a passing fragment after ureteral stent removal, which necessitated placement of a nephrostomy tube. This scenario brings to light several postoperative considerations. First, it reinforces the notion that continued passage of fragments should be expected after stent removal, due in part to the sheer amount of fragments funneling at the ureteropelvic junction. Secondly, the surgeon may underestimate the size of residual fragments during lithotripsy because larger stones could be buried beneath the “gravel” of smaller fragments, therefore increasing the risk of secondary obstruction. For this reason, we feel that consideration should be given to placement of a nephrostomy prior to Stage I as a means of increased renal drainage during staged ureteroscopy, and also in an effort to have readily available renal drainage should infection or obstruction occur after stent removal. Had this been done in our case, we may have been able to avert the final procedure. A last postoperative suggestion would be the gradual weaning of antibiotic therapy for two to three weeks as the patient recovers.

## 4. Conclusion

We feel that the treatment of choice for large staghorn renal calculi remains PCNL. However, in select patients, the literature has shown that a staged ureteroscopic approach can be effective in stones as large as 3 cm. To date, little data exists to support a staged ureteroscopic approach for stones up to 5 cm. In our case, we have demonstrated that with careful surgical planning and efficient surgical technique, even stones up to 5 cm can be treated via a staged ureteroscopic approach.

## Figures and Tables

**Figure 3:**